# I Believe It Is Healthy—Impact of Extrinsic Product Attributes in Demonstrating Healthiness of Functional Food Products

**DOI:** 10.3390/nu13103518

**Published:** 2021-10-07

**Authors:** Brigitta Plasek, Zoltán Lakner, Ágoston Temesi

**Affiliations:** Institute of Economics Science, Department of Food Chain Management, Hungarian University of Agriculture and Life Sciences, Villányi Road 29-43, 1118 Budapest, Hungary; Lakner.Zoltan.Karoly@uni-mate.hu (Z.L.); Temesi.Agoston@uni-mate.hu (Á.T.)

**Keywords:** functional foods, healthy eating, credibility, extrinsic attributes, conjoint analysis

## Abstract

Due to the high proportion of impulse purchases and the short time devoted to purchase decisions, packaging and other extrinsic attributes are becoming increasingly important in demonstrating the health benefits of a functional food item to consumers as plausibly as possible. Our research aims at identifying the role of extrinsic features (claims related to ingredients and health claims, organic or domestic origin, as well as the shape and color of packaging), gathered in the course of in-depth literature analysis, in the case of a functional smoothie. Our online consumer questionnaire was completed by 633 respondents, and the answers were assessed by choice based conjoint analysis. Our results show that each examined attribute plays a role in the assessment of health effects. The color blue has the biggest impact on making the consumer believe in the health benefits of the product. This is followed by the indication of organic origin, then the statement emphasizing the natural quality of the ingredients. The assessment of the specific extrinsic attributes is affected by consumers’ general health interest level, their involvement with food items, and their various demographic features.

## 1. Introduction

A significant proportion of purchases are impulse purchases, and packaging plays a key role in this type of purchase [1,2,3]. Various factors can serve as bases for impulse purchases [4], such as a promotion, personal characteristics, the shop environment, demographic, situational, and social factors [5] and also the perceived health effect of the product. The assessment of the healthiness of food products is a critical factor influencing the success of food-related businesses [6], and for functional foods this effect is of vital importance. A key issue for companies developing functional foods is to be able to use the short purchasing decision situation to show the customer the benefits of the product [7], including its health benefits, as a competitive advantage of these products lies in their additional health benefits on top of the basic nutritional effects [8]. Pramudya and Seo [9] maintain that packaging is one of the most important extrinsic features that influence consumer perception and decision-making. 

Assessing the impact on health is a particularly difficult task for the consumer as healthiness is a kind of credence attribute [10,11,12]. According to Verbeke [13], credence attributes are “product characteristics that can neither be directly perceived nor verified by consumers”. A unique feature of them is that they are difficult to assess even after consumption [14,15]. Nevertheless, the product is assessed as a complex whole, based on intrinsic and extrinsic attributes [16,17]. Research has shown that consumer assessment of the health impact of a product is influenced by several extrinsic [18] and intrinsic attributes [19], as well as non-product-specific factors: e.g., prior knowledge of the consumer [20,21,22]. Such an intrinsic factor is the various ingredients found in a product [23,24,25,26,27,28,29] as well as the taste and other sensory features of the product [30,31,32,33,34]. At the same time, consumers tend to use extrinsic characteristics instead of other factors as an indicator of product quality [35] and they have to rely on them in a shopping environment. The first purchase made by a consumer also heavily depends on these extrinsic attributes [36,37]. Extrinsic features are important in that they allow the product to adapt to changing consumer needs without the manufacturer making any changes to the product itself [19,38].

In addition to playing an important role in the assessment of product quality, extrinsic cues are also features that significantly influence consumer decision-making, and help the assessment of the expected performance, safety, and social acceptance of the product [39], and their role is also important when examining perceived healthiness as well [40,41]. 

## 2. Aim of Research

Our research aim is to examine which extrinsic features, when combined, result in a product that most plausibly shows the consumer that it has a beneficial impact on health. We included attributes based on our literature reviews [42,43]: we examine various claims related to ingredients, health claims, the effect of organic and domestic (Hungarian) origin, as well as the effect of the shape and color of packaging. While our aim was to identify a packaging combination that would convincingly show a health effect, we also aimed to determine the weight of the different factors and characteristics identified by previous research and to rank them in order of importance. Although previous research has already looked at the factors that we are now ranking, we thought it was important that we also investigated which factors are most important in determining the health impact of the functional test product.

The aim of our research was also to clarify the contradictory findings on the shape and color of packaging in the literature. Based on the results of previous research, the shape of the packaging is also a factor influencing the assessment of healthiness [44,45,46]. The shape of the packaging may indicate the physique desired by the consumer [46], and may influence calorie consumption, and thus the perceived effect on health [47]. In addition to the humanoid form, square and rounder packaging were foregrounded in several studies [48,49,50]; however, their results hardly provide a basis to identify a shape which shows consumers the most that the product is beneficial to health. 

In addition to the shape of the packaging, its color is also a factor influencing perceived healthiness [50,51,52,53,54]. According to several studies, the colors blue and green make a positive contribution to the assessment of the healthiness of a product [51,54]. Some results suggest that the color red conveys a kind of prohibition to consumers [52], it does not make them feel that the product is healthier, rather, it makes them think that the product is unhealthy. According to the research results of Huang and Lu [51], respondents perceived a blue-packaged product to be healthier than a red-packaged one. At the same time, for certain products the color red still contributes to the assessment of perceived healthiness [50,54]. 

Our research also aimed to assess the differences between consumer groups in terms of their perception of health impacts. Research has highlighted the relationship between consumer characteristics (gender, age, level of education) and the assessment of functional foods [55,56,57]. Several studies have concluded that women are more open to functional foods than men, and those with a higher education level and the older age group have a completely different attitude towards the perception of functional foods than those with a lower education level or those who are younger in age [56,57,58,59,60,61]. Several previous studies examining purchase intention for functional foods foreground consumers’ health awareness, attention to health and health motivations among the influencing factors [62,63,64,65,66]. This research repeatedly highlights the important impact of consumer health motivations, thus for example Bornkessel, et al. [67] pinpoint health motivations as the factor which most influences awareness of functional food ingredients. In addition to the importance of health motivations; Steinhauser, Janssen and Hamm [66] also found that consumers with higher health motivation spent more time studying health claims and nutrition claims, but it did not affect their purchase intention. Our research aim is related to consumers’ health interest, as measured by the items of the General Health Interest Scale constructed by Roinien and colleagues [68].

In addition to influencing the purchase intention of functional foods [69], food involvement also plays a role in the assessment of the healthiness of different products [70]. Although indirectly, the different levels of food involvement contribute to consumers’ food choices, including how healthy the chosen products are [71]. Involvement, however, contributes not only to the different assessment of perceived healthiness, but also to the processing of nutritional information [72] and to the different utilization of extrinsic features, for example. For consumers with lower involvement, extrinsic features facilitate a simplified assessment process [73]. To measure food involvement, we used the items of the Food involvement scale developed by Brunsø, et al. [74], which is an element of the modified food-related lifestyle model.

### 2.1. Hypothesis Building

#### 2.1.1. The Role of Claims Related to Ingredients, Health Claims and Nutrition Claims

In our research, we examine whether the highlighting of ingredients with separate claims has an impact on the assessment of health effects; we also examine the influence of the natural quality of an ingredient, the added protein content, or the high vitamin content on consumers. 

Research shows that consumers consider natural foods healthier than processed products [75,76]; also, the naturalness of a product is an indicator of perceived healthiness for consumers [21]. Wąsowicz, Styśko-Kunkowska and Grunert [54] maintain that consumers consider healthy products to be natural, among other things. Related to the natural character of a product, we examine the effect of the claim “with natural ingredients.”

The two other claims we examine in our research relate to vitamin content and protein content. The research results of Rizk and Treat [29] show that in addition to other ingredients, some consumers are influenced by the protein content of a product in the perception of its healthiness, while others ignore protein and other ingredients when making a decision [24]. To assess the significance of this ingredient, we examine the claim “26 g protein per portion”.

Vitamins and minerals also influence consumers in their assessment of health effects; some consumer segments specifically prefer foods rich in vitamins and minerals [23]. We examine the effect of emphasizing vitamin content through the claim “rich in vitamin C”, which is a claim widely used by manufacturers in the soft drinks market.

Health claims and nutrition claims are important influencing factors in helping consumers to assess health effect [51,77], though Orquin and Scholderer [78] showed that health claims have only a small impact on the assessment of health effect. Using such claims can greatly influence consumers in their perception of a product [79], although certain consumer groups are skeptical about them and ignore them [80]. Research related to health claims has examined the effect of various health claims on the assessment of the healthiness of a product [81], how credible they are and in what form they are credible to consumers [82,83,84].

Research on ingredients has shown that consumers mostly pay attention to the product ingredients that nutritionists emphasize in relation to a healthy diet, such as the content of sugar, salt, fat and omega-3 fatty acids [26,27,28,29,85]. Health claims also play an important role in the perception of healthiness [86]; at the same time, too much information can make consumers skeptical [87,88], making the role of health claims questionable. Based on the results of several studies, we can state that using nutrition claims and health claims will make the consumer perceive the product as more beneficial to health [89,90,91,92], although skepticism arising in consumers [93] may offset this effect. Thus, we assume that health claims will have a positive effect on the perceived healthiness of a product, although to a lesser extent than the other examined characteristics. Based on all this, our first hypothesis is the following:

**Hypothesis** **1** **(H1).** A claim related to an ingredient has a stronger influence on the assessment of the health benefits of a food product than displaying a health claim.

#### 2.1.2. Organic Origin

According to the results of several studies, the organic origin of products has a positive effect on the assessment of the healthiness of a product [25,94,95,96,97]. Health-conscious consumers also tend to be more open to organic food while typically ignoring the health-related messages of functional food products [80].

**Hypothesis** **2** **(H2).** Of all the factors examined, the organic origin will have the strongest impact on perceived healthiness.

#### 2.1.3. Domestic Origin 

Information on the place of origin of a food item may play an important role in consumer decisions and the assessment of the product [98,99]. Puduri, et al. [100] maintain that consumers prefer information on country of origin because they are concerned about the health effects of foreign products. If the consumer’s perception of a given country is positive, it also affects the perception of the product from there [101]. Previous research has also shown a relationship between country of origin and the assessment of health effect. It is both a significant influencing factor for foods in general [25], and specifically for functional foods [102]. In our fifth hypothesis we assume that although domestic origin has a positive effect on the assessment of healthiness, it is not the most significant factor.

**Hypothesis** **3** **(H3)** . Information on domestic origin has a positive effect on the assessment of the health benefits of a product.

## 3. Materials and Methods

### 3.1. Data Collection

Our data collection methodology was an online consumer survey, which yielded 633 respondents between November and December 2020. Data collection took place on the university’s social media interface through paid advertisement. Respondents provided written consent for their answers to be analyzed. The distribution of the sample by demographic and other characteristics is shown in Table 1.

A big advantage of online sampling is time- and cost-effectiveness [103]; yet, it also involves drawbacks, such as lower response rate or non-representative samples [104]. Our research did not aim at a representative sample, and as a result of online sampling, the distribution of the respondents is biased in several respects, such as the respondents’ education or gender. 

The questionnaire used in the consumer survey can be divided into three main parts. In the first part, we asked respondents to choose between the products with different designs. Then, we asked respondents to evaluate attitude statements which later provided a basis for differentiating between the individual consumer groups. The claims related to healthy lifestyle were measured using elements of the General Health Interest scale [68], whereas food-related consumer involvement was measured using the corresponding scale of the Food related lifestyle model [74]. The third part of the questionnaire included demographic questions.

### 3.2. Choice-Based Conjoint Analysis

Conjoint analysis is a widely used method in behavioral research [105], used, among others, to assess consumer preferences. To achieve our research aim, we performed choice-based conjoint analysis, during which we showed respondents choice-sets with two product combinations each, from which they could choose one, simulating a scenario close to a real choice situation [106]. 

**Figure 1:**
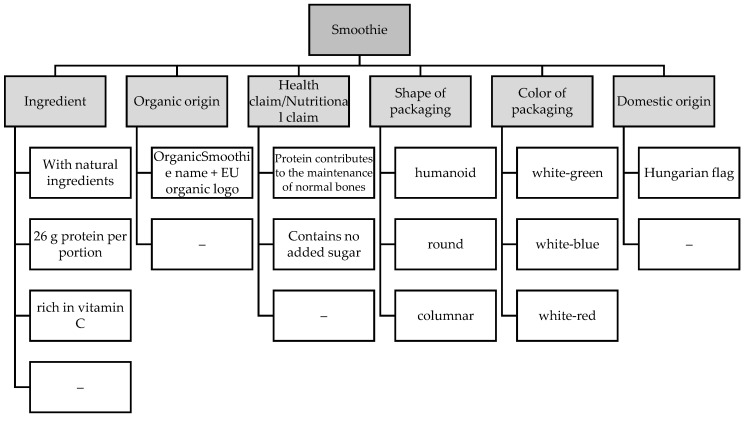
Summary of attributes and levels.

To examine the individual levels, we used a smoothie product for several reasons. The market for functional drinks has been increasing in recent years [107], and smoothies have become an alternative to healthy eating for consumers [108]. In our analysis, we examined the effects of 6 attributes: claims related to ingredients (4 levels), organic origin (2 levels), health claims (3 levels), shape of packaging (3 levels), color of packaging (3 levels) and domestic origin (2 levels). These factors are based on two in-depth literature reviews, which revealed several product-specific and non-product-specific characteristics that influence functional food-related credibility and the assessment of the health effects of a product [42,43]. When completing the questionnaire, respondents always had to choose the picture which they thought presented a product more beneficial to health. The questionnaire did not have a no choice option. Figure 1 summarizes the attributes and their levels.

To create the choice sets, we used Aizaki and Nishimura’s [109] 5-step description, and based on this, the R statistics software [110]. Accordingly, we first created full factorial design with the help of the AlgDesign package. However, given the extremely large number of combinations thus obtained (4 × 2 × 3 × 3 × 3 × 2 = 432), we used orthogonal design, which allows for the examination of the main effects without having to examine all the combinations that exist [111]. Accordingly, 16 combinations were used in the next steps, in 16 choice sets, comparing two product combinations in each case.

Random utility theory states that consumers make rational decisions, maximizing the utility of their decisions. According to the theory, perceived utility (*U_j_*) can be divided into two parts, systematic utility (*V_j_*), and a random component (*ε_j_*) [112], and can be described with the following equation:

*U_j_ = V_j_ + ε_j_*

Based on the attributes and levels used in our research, the representative component of utility can be described using the following equation:

*Vj = ß_I_Ing_j_ + ß_O_Org_j_ + ß_H_Hcl_j_ + ß_S_Sha_j_ + ß_C_Col_j_ + ß_Ori_Ori_j_*

where, *V_j_* is the representative component of utility in the case of *j* smoothie (*j = A,B*, *A—option 1, B—option 2*), the value of *Org_j_* is 1 if an organic product features in the given *j* combination; if not, then it is 0. The value of *Ori_j_* is also 1 if an indication of Hungarian origin appears in the given *j* combination; if not, then it is 0. *Ing_j_*, *HCl_j_*, *Sha_j_*, *Col_j_* indicate a claim related to an ingredient, a health claim, shape of packaging and color used with *j* smoothie. *ß_I_*, *ß_O_*, *ß_H_*, *ß_S_*, *ß_C_*, *ß_Ori_* are unknown parameters associated with *Ing_j_*, *Org_j-_*, *HCl_j-_*, *Sha_j_*, *Col_j_* and *Ori_j_*.

**Figure 2:**
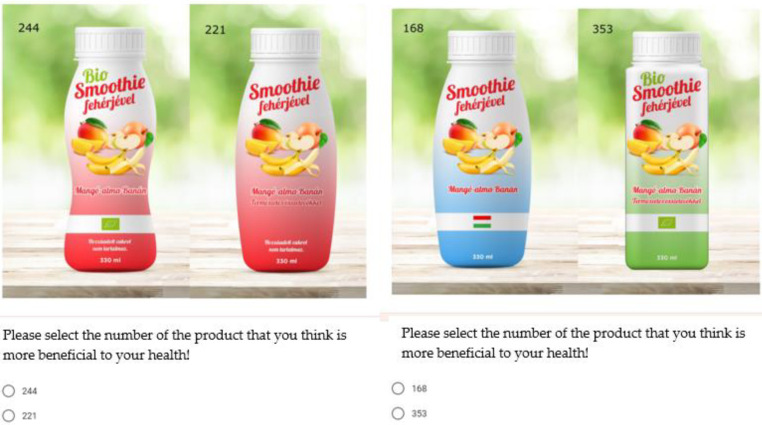
Examples of choice sets.

Figure 2 provides an example of the choice sets as they appeared in the online questionnaire. 

## 4. Results

Our main research aim was to identify the extrinsic attribute combination which results in a product that most convincingly shows the consumer that it contains a beneficial impact on health. We summarize the results of the conditional logit model analysis for the whole sample in Table 2. In the model, the last category of each attribute is a reference category with a coefficient value of 0, so they do not appear in the table.

As the results in Table 2 show, each attribute contributes to the assessment of the health effect of the product to some extent and there was a significant feature for each. Considering the obtained coefficients on the whole, the assessment of health effect is most supported by the color white-blue, as well as organic origin of the product. Based on this, we were able to partially verify our second hypothesis, as the second most influential factor is organic origin.

In our research, we examined different types of claims, such as claims related to ingredients and health claims. The use of all ingredient claims helps to assess the health effects of a product. However, whereas the use of the claim “26 g protein per portion” increases the degree of credibility by 1.3 times (Exp coef = 1.32), and the claim “rich in vitamin C” by 1.6 times (Exp coef = 1.6), the claim “with natural ingredients” doubles it (Exp coef = 2.01) compared to not displaying such a claim. Our results confirm our first hypothesis, in which we assumed that the use of ingredient claims makes the health effect more credible than health claims.

Scrutinizing health claims and nutritional claims, not all examined factors showed a significant effect. Whereas the applied nutritional claim (“Contains no added sugar”) contributes to a more authentic demonstrations of the health benefits of the product, the effect of the examined health claim is not significant. When displaying this nutritional claim on the packaging, consumers are 1.7 times more likely (Exp coef = 1.69) to consider a product beneficial to health than without such a claim on the packaging.

Examining shape, we concluded that using the columnar shape is the most advantageous, while there is no significant difference between the assessment of the health effect of the round and humanoid shape. If instead of the humanoid shape, the manufacturer uses the columnar shape to package a functional smoothie, consumers are 1.4 times more likely (Exp coef = 1.37) to assess the product as beneficial to their health. If the manufacturer uses the color white-blue instead of white-red as the emphasized color of the packaging, it is four times (Exp coef = 3.99) as likely that the consumer will consider the product to be beneficial to their health as if the manufacturer had used the color white-red. This ratio is also significant in the case of the color white-green, where the consumer is nearly twice as likely (Exp coef = 1.87) to assess a white-green-packaged functional smoothie to be beneficial to health than a white-red one. 

An indication of domestic origin also makes the health benefits of a product more credible, which confirms our third hypothesis. A functional smoothie with an indication of domestic origin on the packaging is nearly twice as likely (Exp coef. = 1.83) to be perceived by the consumer as beneficial to health than a product without such an indication. 

Based on the results in Table 2, the product combination considered to be the healthiest is the one that is organic, white-blue in color, includes the statement “with natural ingredients”, an indication of domestic origin, a nutritional claim, and is square shaped.

### The Effects of Individual Characteristics on Valuation

In addition to surveying the whole sample, we assumed that the different characteristics of consumers would result in differences in the assessment of the individual levels. We examined the influence of consumers’ general health interest level, involvement with food, and the different demographic features on the assessment of healthiness. We assessed the differences between women and men, those with higher and lower level of education, and those aged under 36 and those 36 years old or older. When examining General Health Interest, we split the sample into two: respondents with a below average and those with an above average GHI level, based on averaging the values given to the scales. Based on the mean values, we divided the sample into two parts with roughly equal number of elements, then coded it into the table used for the conditional logit model with codes 0 and 1. The code 0 indicated a below average GHI level, and 1 indicated an above average 1. We also proceeded similarly with involvement.

Based on the description of Aizaki and Nishimura [109], we supplemented the command line run on the whole sample in R with the various criteria and examined the significant discrepancies. The results thus obtained are summarized in Table 3, in which the rows where we found a significant difference are highlighted.

According to the gender, age, and education of respondents, we found significant differences at several points.

The gender of the respondent influences the assessment of the different levels for two of the six attributes. Women assess the health impact even more credible than men if the manufacturer uses columnar packaging instead of humanoid (Exp coef = 1.27), and women also ascribe greater importance to the statements “Rich in vitamin C” (Exp coef = 1.5) and “With natural ingredients” (Exp coef = 1.29).

The age of the respondent gains importance in relation to health claims. Respondents under the age of 36 are more likely to believe the health benefits of a smoothie containing either a nutritional claim or a health claim than the older age group (Exp. coef = 1.65; 1.32). Education plays an important role in the case of two ingredient claims and a shape. As opposed to the manufacturer not using such a claim, respondents with a higher education judged the claims “With natural ingredients” and “26 g protein per portion” equally (Exp coef = 1.29 in both cases) more useful when assessing the impact on health than those with a lower education. On the other hand, compared to humanoid packaging, respondents with a higher education are less likely to believe that a product with a round shape packaging is beneficial to health (Exp coef = 0.79) than those with a lower education. 

We obtained interesting results related to organic origin. Consumers with a higher general health interest are less likely to believe that an organic product is beneficial to health than those with less such interest (Exp coef = 0.88). Furthermore, those with a higher food involvement level are more likely to consider an organic functional smoothie beneficial to their health than the less involved (Exp coef = 1.239). Those with a higher general health interest also assessed the round shape differently: compared to a humanoid shape, they consider it less credible (Exp coef = 0.87) that a product with a round shape is beneficial to health than respondents with a lower GHI level.

## 5. Discussion

Extrinsic cues play a prominent role in the assessment of a product. Although the effect on health is a kind of credence attribute, its assessment is strongly influenced by extrinsic product features. In our research, we aimed to determine the extent to which different characteristics used on the packaging influence the consumer assessment of the health impact of a product. Another aim of ours was to clarify the conflicting results found in the literature regarding the shape and color of the packaging. We examined six extrinsic attributes: claims related to ingredients, organic origin, health claims, the shape and color of packaging, and domestic origin. Our results show that of the extrinsic characteristics examined, it is the color white-blue followed by organic origin that have the greatest effect on the consumer’s belief in the health benefits of a product.

By showing that the color white-blue has the strongest influencing effect, we confirm the results of previous research [54] that also highlight this color, with the addition that white-blue contributes four times more effectively to the assessment of healthiness than white-red. This is supported by the results of research by Reutner and his colleagues [52], which demonstrates that the use of red tends to influence consumer perceptions of products that are perceived as unhealthy. Thus, we can rather support the prohibitive nature of the color red. Organic origin should be highlighted in the sense that, although previous research has consistently shown it to be a strong factor influencing perceptions of health impact [25,94,95,96,97], it has not been the most important factor in our case. The fact that organic origin is the second most influential factor is presumably due to the health halo effect associated with it, which makes consumers perceive such products as healthier [113,114]. These two factors are followed by a claim related to ingredients (With natural ingredients) and the indication of domestic origin. That the perception of products is influenced by the inclusion of information on domestic origin has been confirmed by several studies [98,99,100,101,102], and the results of our research contribute to this by ranking the importance of this information. In the order of importance, health claims/nutritional claims are only the fifth of the six elements, and only the nutritional claim showed significant effect, the tested health claim did not. We confirm the results of previous research that consumers may be skeptical about these claims [54,80,93] and also supports the findings of Orquin and Scholderer [78] that health claims have only a small influence on perceptions of healthiness. The form of packaging is also a factor that has been studied in several previous studies [46,47,48,49,50], but its importance in assessing the credibility of the health impact is, based on our results, small compared to the factors studied. Shape has the least influence on the assessment of healthiness, and although previous research has suggested the use of a rounder shape [50], our result suggest that the square shape is more effective.

The non-significant effect is probably due to consumer skepticism, confirming previous research results which showed that consumers may become skeptical about health claims [80,93]. Regarding color, in line with some previous results [51,54], the significant effect of the color white-blue can be highlighted, which, of the examined colors, can contribute the most to consumer belief that the product has a beneficial effect on health.

In addition to the fact that the manufacturer should pay attention to these differences between target groups, we also conclude that the most prominent factor, the color white-blue, positively influences the assessment of the effect on health regardless of the examined consumer criteria and attitudes, and we can draw a similar conclusion in relation to the indication of domestic origin.

At the same time, it is important that manufacturers know their target groups, as the assessment of further attributes varies depending on involvement with food, the level of general health interest, and the different demographic criteria. We found significant differences between consumers with below average and above average general health interest, and between consumers with different involvement levels. When assessing healthiness, organic origin is more important for consumers who are involved above average than for those below average. Claims related to ingredients were assessed differently by women and men, and by respondents with lower and higher education: women are more likely to believe that a smoothie containing the claim “With natural ingredients” or “Rich in vitamin C” is beneficial to health than men. Furthermore, the same is true for those with a higher education rather than a lower education for the former claim or the claim “26 g protein per portion”.

Our results are useful to companies producing functional foods, because in addition to collecting the main extrinsic characteristics that affect perceived healthiness, we also determined their weight. It all helps manufacturers to most effectively imply the healthiness of a product, which is of great relevance in the case of functional foods, to consumers during the purchasing process. A functional food producing company’s marketing strategy must consider the health benefits of its products, among many other aspects. If a company knows the strength of each of the factors that significantly influence health impact, it can make better decisions about which aspects to change even though they may require changes to other elements of the marketing strategy, and which aspects to leave unchanged because of other factors influencing the marketing strategy. 

Our results show that it is not the nutritionally valuable sources of information that most influence consumers’ perceptions of product healthiness, which raises the importance of the work of nutritionists and dieticians. As credible communicators [115], they have an important role to play in ensuring that health-conscious consumers are more conscious when making a purchase, and seek and evaluate the right information.

## 6. Limitations

In accordance with the Total Food Quality model of Grunert, et al. [116], which treats price as a separate category outside of extrinsic features, we did not explicitly include price among the examined factors in our research. However, price can be a significant influencing factor, so it would be worth exploring this in a future study.

Our research is based on two in-depth literature analyses, but it is important to note that the factors under investigation, such as color, can also have other meanings. The two reviews focused specifically on perceived healthiness and this research was not designed to explore other present-tense aspects.

## 7. Conclusions

In our research, following an analysis of the literature, five characteristics that influence the perception of a product’s health impact have been identified, namely the shape and color of the packaging, health claims, claims related to the ingredients of the product and the impact of domestic origin. Our aim was to rank the characteristics in order of which most influence whether a consumer believes a functional food to be beneficial to health. We also aimed to determine the weight of different characteristics in the assessment of health impact. According to our results, among all the six examined attributes there are characteristics which significantly influences the credibility of the health effect. Our results show that consumers are most likely to believe that product is beneficial to their health if it is primarily white and blue, organic and contains an ingredient claim. These are followed to a lesser extent by the indication of domestic origin and the nutritional claim, and least influenced by the form of the packaging. However, we found that in the perception of health effect even the shape that resembled the humanoid shape differed significantly from the columnar shape. In addition, we consider it an important part of our results to point out that while health claims do not significantly affect the credibility of the health effect, nutritional claims do. The smoothie with the simplest packaging was the least likely to be perceived by respondents as having health benefits. This means that consumers were least likely to believe that the packaging was beneficial to health if it was red-white, not organic, did not contain any ingredient claims or health claims, did not have a domestic origin label and was angular in shape.

In the functional food market, a significant proportion of products are withdrawn by companies shortly after launch. The results of our research may help manufacturers to create and present packaging in a combination that consumers are more likely to believe has positive health benefits.

Although our research results have shown which features contribute the most to making the consumer believe that a product has a beneficial effect on health, the question arises whether the combined use of so much information would be good corporate practice. It is possible that packaging with much less information more effectively presents the healthiness of the product to the consumer. Further research may aim to gauge how much information a manufacturer should use on the packaging to convey a sufficiently credible effect on health to the consumer.

## Figures and Tables

**Table 1:** Respondents’ demographic and other characteristics (*n* = 633).

Variables		Sample Composition
		%
Gender	male	26.2
	female	73.8
Age group	18–25 years	25.4
	26–35 years	19.6
	36–45 years	15.2
	46–55 years	15.2
	56 years and older	24.6
Education	max 8 years of elementary school/trade school/vocational school	9.1
	secondary school diploma	47.6
	higher education degree	43.6
Place of living	Capital	28.4
	Greater capital area	11.2
	Countryside town (not in the greater area)	44.2
	Village/settlement outside of the greater area	16.1
Perceived income status	very tight/tight	21
	average	53.2
	good/very good	25.8
Person responsible for grocery shopping in the household	Respondent	47.4
	Other	6.3
	Shared	46.3
Size of household	1 person	14.1
	2 persons	38.2
	3 persons	21.8
	4 persons	14.5
	5 or more persons	11.4

**Table 2:** Results of the conditional logit model.

Attribute—Claim Related to an Ingredient				
Level of attribute	Coefficients	Exp (coef)	se (coef)	z-value
With natural ingredients ^a^	0.698 **	2.010	0.046	15.168
Rich in vitamin C ^a^	0.469 **	1.599	0.065	7.211
26 g protein per portion ^a^	0.280 **	1.324	0.066	4.254
**Attribute—Organic**				
OrganicSmoothie name + EU organic logo ^b^	1.016 **	2.761	0.033	30.596
**Attribute—health claim**				
With no added sugar ^c^	0.529 **	1.698	0.05	10.476
Protein contributes to the maintenance of normal bones ^c^	−0.087 ^n.s.^	0.917	0.059	−1.458
**Attribute—shape**				
columnar ^d^	0.315 **	1.37	0.053	5.959
round ^d^	0.046 ^n.s.^	1.047	0.048	0.959
**Attribute—color**				
white-blue ^e^	1.385 **	3.992	0.153	9.037
white-green ^e^	0.627 **	1.873	0.089	7.004
**Attribute—origin**				
Hungarian flag ^f^	0.606 **	1.833	0.0572	10.602

^a^—reference category: packaging with no claim on an ingredient; ^b^—reference category—nonorganic product; ^c^—reference category—packaging with no health claim; ^d^—reference category—humanoid shape; ^e^—reference category—white—red; ^f^—reference category—packaging without a Hungarian flag; ** *p* < 0.01, n.s.—non-significant.

**Table 3:** Results of the conditional logit model: Interaction effects.

Attribute—Claim Related to Ingredient					
Level of attribute	Interaction effect	Coefficients	Exp (coef)	se (coef)	z-value
With natural ingredients ^a^	:gender	0.216 *	1.240	0.106	2.021
	:education	0.262 *	1.299	0.135	1.931
Rich in vitamin C ^a^	:gender	0.407 **	1.502	0.149	2.726
26 g protein per portion ^a^	:education	0.262 *	1.299	0.135	1.931
**Attribute—Organic**					
OrgSmoothie name + EU organic logo ^b^	:General health interest	−0.128 *	0.88	0.068	−1.872
	:involvement	0.214 **	1.239	0.069	3.090
**Attribute—Health claim**					
Contains no added sugar ^c^	:age	0.502 **	1.652	0.106	4.719
Protein contributes to the maintenance of normal bones ^c^	:age	0.281 **	1.324	0.125	2.251
**Attribute—Shape**					
round ^d^	:General health interest	−0.131 *	0.877	0.078	−1.669
	:education	−0.229 **	0.795	0.078	−2.937
columnar ^d^	:gender	0.239 **	1.27	0.12	1.996

^a^—reference category: packaging with no claim on an ingredient; ^b^—reference category—nonorganic product; ^c^—reference category—packaging with no health claim; ^d^—reference category—humanoid shape; * *p* < 0.1, ** *p* < 0.05;

## Data Availability

The data presented in this study are available on request from the corresponding author.
